# Profiles of Motor Laterality in Young Athletes' Performance of Complex Movements: Merging the MOTORLAT and PATHoops Tools

**DOI:** 10.3389/fpsyg.2018.00916

**Published:** 2018-06-07

**Authors:** Marta Castañer, Juan Andueza, Raúl Hileno, Silvia Puigarnau, Queralt Prat, Oleguer Camerino

**Affiliations:** ^1^National Institute of Physical Education of Catalonia (INEFC), University of Lleida, Lleida, Spain; ^2^Lleida Institute for Biomedical Research Dr. Pifarré Foundation (IRBLLEIDA), University of Lleida, Lleida, Spain

**Keywords:** laterality profiles, PATHoops (spatial orientation), MOTORLAT (motor laterality inventory), contralateral synergy, complex movements

## Abstract

Laterality is a key aspect of the analysis of basic and specific motor skills. It is relevant to sports because it involves motor laterality profiles beyond left-right preference and spatial orientation of the body. The aim of this study was to obtain the laterality profiles of young athletes, taking into account the synergies between the support and precision functions of limbs and body parts in the performance of complex motor skills. We applied two instruments: (a) MOTORLAT, a motor laterality inventory comprising 30 items of basic, specific, and combined motor skills, and (b) the Precision and Agility Tapping over Hoops (PATHoops) task, in which participants had to perform a path by stepping in each of 14 hoops arranged on the floor, allowing the observation of their feet, left-right preference and spatial orientation. A total of 96 young athletes performed the PATHoops task and the 30 MOTORLAT items, allowing us to obtain data about limb dominance and spatial orientation of the body in the performance of complex motor skills. Laterality profiles were obtained by means of a cluster analysis and a correlational analysis and a contingency analysis were applied between the motor skills and spatial orientation actions performed. The results obtained using MOTORLAT show that the combined motor skills criterion (for example, turning while jumping) differentiates athletes' uses of laterality, showing a clear tendency toward mixed laterality profiles in the performance of complex movements. In the PATHoops task, the best spatial orientation strategy was “same way” (same foot and spatial wing) followed by “opposite way” (opposite foot and spatial wing), in keeping with the research assumption that actions unfolding in a horizontal direction in front of an observer's eyes are common in a variety of sports.

## Introduction

**Figure 1:**
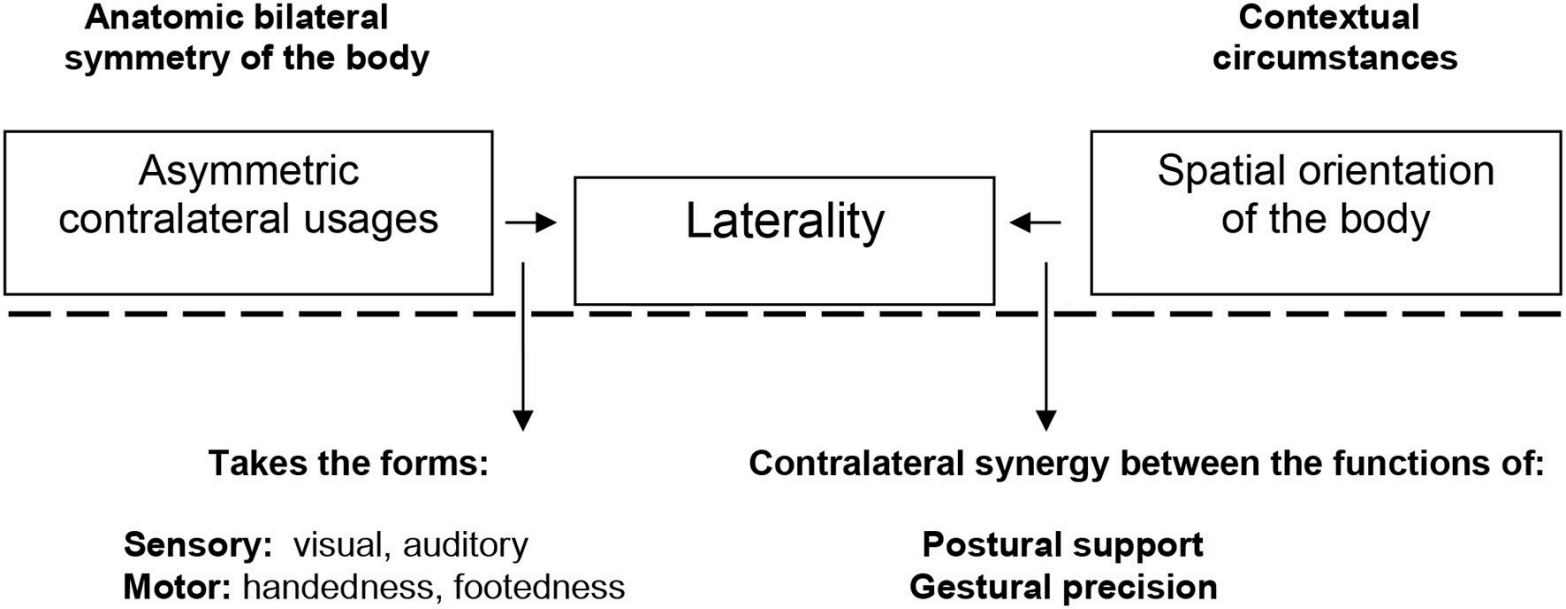
Aspects involved in motor laterality assessment.

Our bodies are able to move among all kinds of surroundings thanks to hemispheric dominance, which, when linked to orientation in spatial contexts, shapes our usage of laterality with regard to our limbs. Thus, as explained in Figure 1 below, the human body is anatomically symmetric (bilateral) but functionally asymmetric (contralateral), depending on its movement needs and contextual circumstances (for a review, see Brancucci et al., 2009). These circumstances give rise to different contralateral usages of the two sides of the body, performed mainly by the limbs during the execution of motor actions that define our personal motor laterality profiles. In recent years, a growing number of studies have mentioned laterality in relation to technical, behavioral, physical, and tactical factors in sports (Carling et al., 2005; Hodges et al., 2006), but without delving deeper into this subject or offering specific tools.

In this study, we present two tools suited to the study of laterality profiles and spatial orientation that fit with the assumptions of spatial stimulus-response compatibility and ideomotor action in the framework integrating perception and action addressed within the Theory of Event Coding (Hommel et al., 2001). The existing tools for assessing laterality do not take into account this framework of perception-action integration or the polymorphism of laterality usages and therefore are used primarily for studying the functions of the upper limbs (mainly the hands).

### Richness of motor skills and lateralization uses

The laterality of the body underpins all motor skills that allow for the richness of movements in everyday situations as well as in specific contexts such as sports (for a review, see Brancucci et al., 2009; Tran and Voracek, 2016). Indeed, laterality must not be reduced to right- or left-handedness, as it is clear that our bodies perform specific and personal uses of lateralization, thereby defining a varied tapestry of motor laterality profiles. Greater research on laterality terms (Gabbard, 1997; Hart and Gabbard, 1998; Westmoreland, 2016) could help to enhance motor performance in all types of movements involving basic, specific, and specialized motor skills, including the mechanical aspects of a technique—the way in which the skill is performed in terms of the kinetic and kinematic details of the movement involved (O'Donoghue, 2010). The roots of fundamental motor skills—locomotor, stability, and manipulation (Gallahue and Cleland-Donnelly, 2003; Castañer et al., 2009, 2015, 2016a)—lie in the phylogenetic contribution (Anderson et al., 2001) and their singular characteristics depend on ontogeny (Assaiante and Amblard, 1995; Salesse et al., 2005), with each individual being optimally geared to adapt to multifaceted environments (Johnson, 2007) such as the complex and dynamic context of sports.

Given the dynamism and complex nature of sports, motor laterality profiles detected using specific tools are of interest for the purposes of optimizing athletes' performance of complex movements (Loffing et al., 2015), which are built on complex intentional actions (Murgia et al., 2014; Schaefer, 2014). Laterality refers not only to left-right preference (Hagemann, 2009; Teixeira et al., 2011; Bishop et al., 2013) but also to how an athlete orients his or her body spatially (Bishop et al., 2013; Loffing et al., 2015). In this regard, previous research related to football (Castañer et al., 2016a) demonstrated that Lionel Messi—a left-footed player—is a good example of laterality, given that he has achieved some of his best results while playing on the right wing. This study showed that Messi “tends to occupy the right midfield and right wing more often than the other parts of the pitch as he moves toward the goal, as this would logically afford him a better angle from which to shoot with his left foot” (Castañer et al., 2016a, p. 8). Although the richness and diversity of sports are due to the high complexity of the athletes' body movements and the contexts in which they perform, general research has exhibited certain flaws: (a) a lack of specific practical tools for observing and detecting a broader range of motor laterality profiles; (b) the simplification of the broad range of right-left and ambidexterity profiles; and (c) a failure to take into account fundamental factors such as spatial orientation and the complementary functions of postural support and gestural precision of the limbs.

### Mastering contralateral body synergy: merging gestural precision and postural support

Specific studies from the late 1980s and 1990s (Peters, 1988a; Previc, 1991; Coren, 1993; Hart and Gabbard, 1998) noted that laterality is described in a bilateral context in which the role of one limb is to execute an action while the role of the other limb is to establish postural stabilization. In terms of more detailed motor conceptualization, we refer to these two roles as *gestural precision* and *postural support*, respectively. We argue that it is essential to distinguish between these two functions performed by limbs working harmoniously together in contralateral synergy and underpinning a particular motor laterality profile.

Despite the large amount of scientific literature related to laterality, there is scant discussion of the conceptual basis of the constituent elements of motor actions (e.g., motor skills, perceptual and conditioning capabilities, technique, and tactics) that underpin laterality. Therefore, gestural precision and postural support functions are based on the diverse—and, at the same time, bilateral—structure of our corporeity, which enables us to simultaneously generate bodily gestures (dynamism) and postures (staticity) (Castañer et al., 2010a, 2016b). In fact, postural sequences are nested in each body gesture (Out et al., 1998; Gabbard and Hart, 2002).

Optimizing motor actions involves mastering physical activity practices and, consequently, making effective use of laterality, with the aforementioned contralateral body synergy playing a key role. This is clear in elite athletes such as Rafa Nadal, who trained to reverse his innate manual preference in order to obtain an advantage over his opponents, and Lionel Messi, who, despite being left-footed, signs his contracts with his right hand. In previous research (Castañer et al., 2016a, 2017a), we detected the role played by laterality in Messi's extraordinary goal-scoring achievements. We reported, for example, that his right turn with his back to the rival goal line was directly related to the use of his left leg. While remaining steady on his right leg, he turns his body, thereby allowing his left leg to perform precise actions. In that study, which compared the motor skills performed by Messi and Cristiano Ronaldo, the term *mastering lateral synergy* was used to refer to an athlete's ability to combine the precision of his/her dominant limb with the stability offered by the other, non-dominant limb.

### Limb dominance and spatial orientation

With regard to limb dominance, the scientific literature (Büsch et al., 2010; Edlin et al., 2015) has shown that the inventories used to assess laterality have certain weaknesses, including (a) insufficient differentiation between dominant precision actions (i.e., the foot that kicks a ball) and support actions (i.e., standing on a foot) (Elias et al., 1998; Peters, 1998b; Gabbard and Hart, 2002) and (b) excessive focus on the handedness of human beings (Westmoreland, 2016), to the detriment of other body parts (i.e., Oldfield, 1971; Kelley, 2012; Papadatou-Pastou et al., 2013; Hardie and Wright, 2014). Hence the need for a useful exhaustive inventory designed to assess the laterality of the body as a whole and the versatility and complexity of its motor actions.

Versatility of complex movements in both individual and team sports requires the integration of multiple skills (Bishop et al., 2013) directly linked to motor anticipation (Murgia et al., 2014) and the linkage of behaviors to outcomes for teams and individual athletes (Glazier and Robins, 2013). Complementarily, lateral asymmetry in sports performance is due to greater use of the dominant limb, particularly for complex motor actions such as shooting, and is largely determined by use, habit and technique acquisition (Teixeira et al., 2011; Edlin et al., 2015).

Nevertheless, an exhaustive assessment of laterality must take into account that all kinds of locomotor, stability, and manipulation motor skills are rooted in the acquisition of spatial concepts (Pitchford et al., 2016) such as spatial structuring, organization, and orientation, which are directly related to uses of hemispheric dominance. In any case, this is a complex reality (Edlin and Lyle, 2013) that also fits in with the “moving while perceiving and thinking” line of analysis (Schaefer, 2014). The complex merging of hemispheric dominance and spatial orientation reinforces the framework integrating perception and action that was first addressed within the Theory of Event Coding (Hommel et al., 2001), which aims to improve our knowledge about how complex movements are performed. Furthermore, temporo-spatial information plays a fundamental role in the multifaceted surroundings where complex movements are performed. In this sense, as Murgia et al. (2017) point out, “the combination of temporal information processing and biological movement perception has rarely been addressed by researchers, nevertheless, it represents an interesting research challenge which might reveal how athletes, dancers, and musicians process temporal information related to complex human movements.” We found in previous studies that spatial information also has a strong influence on perceptive-cognitive processes in the performance of motor actions in a range of different groups, including children and adolescents (Castañer et al., 2016c) and adults and the elderly (Alves Franco et al., 2013; Saüch and Castañer, 2014; Castañer et al., 2015, 2017b; Puigarnau et al., 2016). As shown in Figure 1, laterality is not merely a question of handedness or footedness, but a process that develops in conjunction with the way in which our body uses and orients itself in space (Salesse et al., 2005; Castañer et al., 2012a) and emerges as a factor of perceptual-motor experience.

### Laterality as a factor of perceptual-motor experience

Performance in sports depends on specific perceptual or anticipatory skills (Williams et al., 1999; Hagemann et al., 2006) that are directly related to managing spatial circumstances. Expert athletes can predict, for example, the direction of an opponent's action earlier and more precisely than novices (Hagemann, 2009) and are more skilled at anticipating actions (Chi, 2006; Hodges et al., 2006). Recent studies on perceptual-motor experience in the mastery of various sports (Murgia et al., 2014, 2016; Woods et al., 2014; Pizzera and Hohmann, 2015; Castañer et al., 2016a, 2017a; Camponogara et al., 2017; Sors et al., 2017) have shown that the observer's perceptual-motor experience is a crucial factor for accurate perception of biological movements (Calvo-Merino et al., 2006; Schütz-Bosbach and Prinz, 2007; Murgia et al., 2016). Research in this line using point-light displays has demonstrated how the observer's perception system fits with the kinematic parameters in specific contemporary dance actions (Castañer et al., 2012a; Torrents et al., 2013) and shown how accuracy can be recognized in spatial representation (Fumarola et al., 2016). Likewise, in a recent study (Castañer et al., 2017b), we found, through a mixed methods research analysis (Anguera et al., 2012, 2017), unexpected interpersonal heart rate synchrony between participants during motor-cognitive tasks, which could be related to the cue factors of the Theory of Event Coding: codes (cognitive structures) and sensorimotor synchronization. Perceptual-motor experience implies the enhancement of cognition (Kenny et al., 2016), but traditional approaches tend to consider cognitive and motor skills in isolation, thus preventing the adoption of an integrative approach. In fact, the ability to efficiently and effectively execute skilled movement patterns—which requires the application of cognitive and motor skills to rapidly changing situations—is the most important aspect of an athlete's performance (Ali, 2011).

### The present study

On the basis of the theoretical underpinnings set out above, in the present study we determined how young athletes approach a novel perceptual-motor situation by studying their contralateral uses of the limbs and spatial orientation during the performance of tapping locomotion skills. In parallel, we determined the athletes' laterality profiles by asking them to perform 30 motor skills of increasing complexity that underpin all sorts of complex movements (Camerino et al., 2012). The items were correlated in order to guarantee a perception-action way of detecting these profiles.

With this procedure, we went beyond the traditional procedures for detecting laterality (for a review, see Edlin et al., 2015), which were established on the basis of the terms left-handedness and right-handedness as they are used in sport sciences (for a review, see Tran and Voracek, 2016). Instead, we used the term *motor laterality profile*—right, left, or mixed—which encompasses the whole body, taking into account the lateral synergy that merges postural support and gestural precision (Castañer et al., 2017a).

In sum, we believe that the determination of laterality profiles should include a more detailed study of laterality in relation to the performance of the fundamental and specific motor skills that make up complex movements. Thus, the overall objective of this study was to obtain a broad view of motor laterality profiles by applying two complementary instruments, one which analyzes the contralateral distribution of postural support and gestural precision in a broad spectrum of motor skills (from simple to complex), and another which allowed us to detect spatial orientation by presenting participants with a novel motor situation that activated an ideomotor action as an empirical domain of the perception-action integration framework.

## Materials and methods

### Participants

A total of 95 young athletes (73 males, 22 females) ranging in age from 17 to 26 years (*M*_age_ = 19.7 years; *SD* = 2.01) provided informed consent and participated in the study, which was approved by the ethics committee at the University of Lleida, Spain (code CEIC-1665). Participants were taking part in a program to improve their physical capabilities and motor skills. As part of this program, they signed up for the two tasks included in this study. Participants were required to have practiced their sport—by training or competing—for at least the previous 6 months. Those who were injured at the time of data collection or in the previous month were excluded.

### Materials

#### MOTORLAT: an *ad-hoc* motor laterality inventory

To detect laterality profiles from motor skills performance, we designed a motor laterality inventory called MOTORLAT (Table 1) as an optimized extension of previous research (Castañer et al., 2012a) and we applied measures of inter-rater agreement. MOTORLAT comprised four criteria based on the motor skills-related criteria from the Motor Skills Observation System (OSMOS) instrument (Castañer et al., 2009, 2012a). These four criteria were as follows: (1) *locomotion skills*, referring to actions that require the body to travel from one point to another across space; (2) *stability skills*, referring to actions that do not require the body to travel from one point to another across space (i.e., jumping, balancing, and turning); (3) *manipulation skills*, referring to actions that require the manipulation of objects or other people with the limbs of the body; and (4) *combined skills*, referring to actions that combine one or more of the aforementioned criteria. Each criterion was expanded to build an exhaustive and mutually exclusive total of 30 items of fundamental and combined motor skills (12 related to the lower limbs, 9 related to the upper limbs, and 9 related to the direction taken to execute an action). Moreover, next to each item, there was a clear description of the aspect to evaluate and the boxes for left and right were arranged intuitively for the observer.

**Table 1:** MOTORLAT motor laterality inventory.

	**Motor skill**	**Description**	**Aspect to evaluate**	**Left**	**Right**
**LOCOMOTION**					
1	Sequential	Walks forward from a standing position with feet parallel to each other	Foot used to take the first step	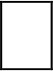	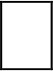
2	Start/stop	Walks around an obstacle from a standing position	Direction taken to walk around the obstacle	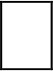	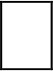
3	Sequential	Walks up steps/stairs from a standing position	First foot used to go up steps/stairs	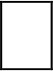	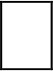
4	Start/stop	Pushed from behind when standing with feet parallel	Foot moved to regain balance	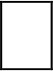	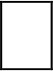
5	Simultaneous	Gets up to walk from a crawling position	Hand moved first	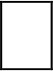	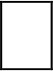
6	Simultaneous	Gets up to walk from a crawling position	Foot moved first	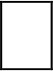	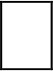
**STABILITY**					
7	Support	Simultaneously raises hand and foot while on all fours	Hand raised	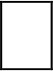	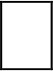
8	Support	Simultaneously raises hand and foot while on all fours	Foot raised	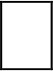	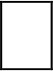
9	Support	Stands on one leg from a standing position with feet parallel to each other	Leg raised	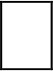	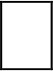
10	Axial	Makes a full turn on both feet from a standing position with feet parallel to each other	Direction of turn	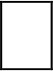	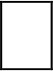
11	Axial	Turns over when lying face up	Direction of turn	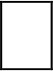	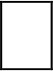
12	Axial	Gets up from a chair and turns around the chair	Direction of turn	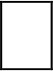	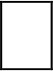
13	Axial	Pivots (turns) on one foot from a standing position with feet parallel to each other	Direction of pivot	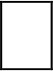	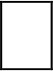
14	Axial	Pivots (turns) on one foot from a standing position with feet parallel to each other	Leg raised during pivot	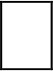	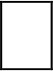
15	Stop	Hops several times from a standing position with feet parallel to each other	Foot raised	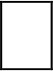	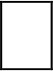
**MANIPULATION**					
16	Impact	Raises arm to touch elevated ball from a standing position with feet parallel to each other	Hand used to touch the ball	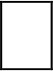	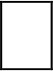
17	Touch/move	Picks up ball from the ground with one hand from a standing position with feet parallel to each other	Hand used to pick up the ball	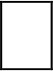	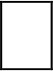
18	Impact	Kicks ball with one foot from a standing position with feet parallel to each other	Foot used to kick the ball	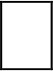	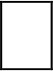
19	Touch/move	Bounces ball with one hand from a standing position with feet parallel to each other	Hand used to bounce the ball	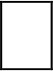	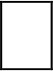
20	Touch/move	Receives ball with just one foot from a standing position with feet parallel to each other	Foot that touches the ball first	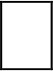	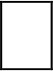
**COMBINATIONS**					
21	Touch/move and axial	Holds ball with one hand in front of face and rotates it around head, switching hands	Direction of rotation	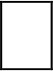	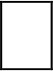
22	Touch/move and axial	Holds ball with one hand in front of face and rotates it around head, switching hands	Hand used to start the movement	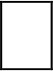	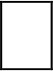
23	Touch/move and axial	Holds ball in front of bellybutton and rotates it around waist, switching hands	Direction of rotation	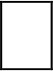	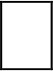
24	Touch/move and axial	Holds ball in front of bellybutton and rotates it around waist, switching hands	Hand used to start the movement	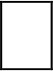	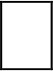
25	Touch/move and axial	Positioned on the floor, uses hand to rotate ball on the ground	Direction of rotation	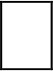	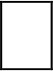
26	Touch/move and axial	Positioned on the floor, uses hand to rotate ball on the ground	Hand used to start the movement	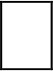	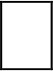
27	Stop and axial	Jumps and turns on one foot from a standing position with feet parallel to each other	Direction of turn	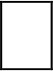	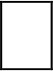
28	Stop and axial	Jumps and turns on one foot from a standing position with feet parallel to each other	Foot raised	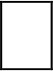	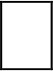
29	Sequential, stop and impact	Sprints from a standing position with feet parallel and then jumps on one foot to touch an elevated object	Hand used to touch the object	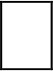	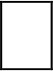
30	Sequential, stop and impact	Sprints from a standing position with feet parallel and then jumps on one foot to touch an elevated object	Foot raised	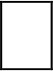	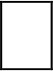

#### PATHoops: the precision and agility tapping over hoops task

**Figure 2:**
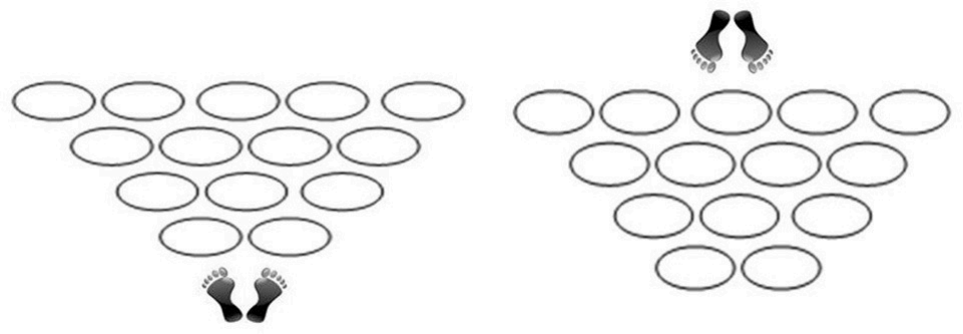
**(Left)** Starting position from the narrow side of PATHoops. **(Right)** Starting position from the wide side of PATHoops.

Precision and Agility Tapping over Hoops (PATHoops) consisted of the following task. Participants, standing on both feet, were asked to perform a path by stepping in each of 14 hoops arranged in a triangular shape on the floor. In addition, participants were asked to perform the PATHoops task from both sides (Figure 2). Performing the task from both sides is in keeping with the assumption of Loffing et al. (2016) that actions unfolding in a horizontal direction in front of an observer's eyes are common to a variety of sports. To measure PATHoops, the researchers recorded the strategies used by the participants: (a) Same way: The athlete goes to the same wing as the foot used in the first step (e.g., right-right); (b) Opposite way: The athlete goes to the opposite wing as the foot used in the first step (e.g., left-right); or (c) Other: The athlete performs some other type of spatial orientation strategy. Given that novelty in motor situations involving fundamental acquired skills guarantees a spontaneous stimulus-response, thus preventing the use of automatic or rehearsed responses (Hommel et al., 2001; Castañer et al., 2010b, 2011, 2012b, 2016c, 2017b; Stöckel and Weigelt, 2012; Torrents et al., 2013), we designed PATHoops to be a novel situation involving locomotor skills. We decided to focus on the locomotor skill of walking quickly—i.e., feet-tapping—because this is a fundamental and automatic motor skill and because it involves multisensory information such as vestibular, visual, and kinesthetic information (Hart and Gabbard, 1997; Gabbard and Hart, 2002; Santoro et al., 2017).

### Procedure

Two researchers—experts in physical activity and sports—administered the instruments to all participants one by one in a sports facility divided by a curtain into zone A and zone B. In order to guarantee that none of the participants had worked out immediately before data collection, all participants attended a non-practical session in a classroom at the facility. Participants were called, one by one, into zone A, where a researcher administered the PATHoops task. Once finished, participants proceeded to zone B, where they completed the MOTORLAT inventory under the supervision of a second researcher.

The materials required for the application of MOTORLAT are as follows: a chair, a step or stairs, and a foam ball. Participants indicated their age, gender, and sports specialty on a data entry form. For each participant, the observer administered the 30 items one at a time, in the indicated order, and checked the box corresponding to the limb (left or right) that the participant used to execute the aspect being evaluated. The observers stated the wording of the items loudly and clearly, and materials were provided to the participant as required for each inventory item.

The materials required for the application of PATHoops are as follows: 14 hoops, each measuring no more than 50 cm in diameter, arranged on the floor in the shape of a triangle (Figure 2). Participants had to perform the task twice, first from the narrow side and then from the wide side, allowing the researchers to observe which foot the participant used to start the task, as well as the participant's left-right preference and spatial orientation. The spatial orientation strategies used by the athletes after the first step to complete the PATHoops task are included in the results section.

The researchers did not perform any examples or models of the PATHoops task or demonstrate any of the motor skills included in the MOTORLAT items.

### Data analysis

Firstly, measures of inter-rater agreement with standard errors and confidence intervals were used to validate the MOTORLAT instrument. This validation was carried out by 35 international experts on physical activity and sports. The 30 items were validated (Wongpakaran et al., 2013; Gwet, 2014) using a Likert scale of 1–3 with the following criteria: unambiguity, appropriateness, and relevance (Table 2). Laterality profiles were obtained by means of cluster analysis. As an internal assessment of these clusters, a correlational analysis was carried out for each cluster between the motor skills of the MOTORLAT items. A contingency analysis was used to cross the limb dominance criteria from the MOTORLAT inventory and their relationships with the spatial orientation criteria from the PATHoops task.

**Table 2:** Measures of inter-rater agreement with standard errors and confidence intervals.

						**95% Confidence interval**	
		**Coefficient**	**Standard error**	***t***	***p***	**Lower limit**	**Upper limit**
Unambiguity	Percent agreement	0.7911	0.0185	42.74	0.000	0.7532	0.8289
	Gwet's AC_1_	0.7350	0.0296	24.81	0.000	0.6744	0.7956
Appropriateness	Percent agreement	0.9074	0.0126	71.88	0.000	0.8816	0.9333
	Gwet's AC_1_	0.8980	0.0153	58.69	0.000	0.8667	0.9293
Relevance	Percent agreement	0.7566	0.0201	37.64	0.000	0.7155	0.7977
	Gwet's AC_1_	0.7226	0.0261	27.73	0.000	0.6693	0.7759

*Number of raters = 35; number of items = 30; number of rating categories = 3 (unambiguity, appropriateness, relevance)*.

## Results

In accordance with the declared objectives of the study, our results were as follows: (a) motor laterality profiles were obtained by analyzing contralateral distribution of postural support and gestural precision for a broad spectrum of motor skills (from simple to complex); (b) spatial orientation was detected from a novel motor situation in which participants were asked to activate an ideomotor action. We present our results in three sections: (a) Motor laterality profiles obtained; (b) Related motor skills in lateralization uses; (c) Spatial orientation and laterality profile.

### Motor laterality profiles obtained

Laterality profiles were obtained by means of cluster analysis and subsequent correlational analyses were carried out. Cluster analysis showed that the criteria of locomotion, stability and manipulation reveal clear motor laterality profiles (Table 3): (1) ambidexterity (1%), (2) left laterality (6%), (3) right laterality (74%), and (4) mixed laterality (19%) (meaning that the athletes perform locomotion and stability motor skills with the left lower limb and in a leftward direction but perform manipulation skills with the right upper and lower limbs). This mixed laterality is made clear by an in-depth analysis of complex movements—for example, those which include the action of jumping. A cluster analysis of jumping skills reveals four profiles (Table 4). Most athletes usually used their right hand to touch an elevated object, orienting their body to the left side. The first profile (cluster 1) corresponds to athletes who raised their right hand and foot during the jump. The second and the third profiles (clusters 2 and 3) indicate an inverse relationship between the right hand and left foot, although the direction of the turn varies. Finally, the fourth profile (cluster 4) corresponds to athletes who raised their left hand and right foot.

**Table 3:** Athlete profiles by dimension of laterality.

	**Cluster 1**	**Cluster 2**	**Cluster 3**	**Cluster 4**
	**Ambidexterity**	**Left laterality**	**Right laterality**	**Mixed laterality**
**LOCOMOTION**				
Foot used to take the first step	0	1	0	1
First foot used to go up steps/stairs	1	1	0	1
Foot moved to regain balance	0	1	0	1
**STABILITY**				
Makes a full turn on both feet	0	1	1	1
Turns over when lying face up	0	1	1	1
Pivots (turns) on one foot from a standing position with feet parallel to each other	1	1	1	1
**MANIPULATION**				
Hand used to touch an elevated ball	0	1	0	0
Hand used to pick up the ball	2	1	0	0
Foot used to kick the ball	0	1	0	0
Hand used to bounce the ball	2	1	0	0
Foot that touches the ball first	2	1	0	0
Number (%) of athletes	1 (1%)	6 (7%)	70 (72%)	18 (19%)

*Cluster analysis showed that the criteria of locomotion, stability and manipulation reveal clear motor laterality profiles: (1) ambidexterity (1% of participants), (2) left laterality (6%), (3) right laterality (74%), and (4) mixed laterality (19%)*.

**Table 4:** Laterality profiles and jumping skills.

	**Cluster**			
	**1**	**2**	**3**	**4**
Hand used to touch the object	0	0	0	1
Direction of turn with feet parallel to each other	1	1	0	1
Foot raised with feet parallel to each other	0	1	1	0
Foot raised to touch an elevated object	0	1	1	0
Number of athletes	56	17	17	5

*The first profile (cluster 1) corresponds to athletes who raised their right hand and foot during the jump. The second and the third profiles (clusters 2 and 3) indicate an inverse relationship between the right hand and left foot, although the direction of the turn varies. Finally, the fourth profile (cluster 4) corresponds to athletes who raised their left hand and right foot*.

### Related motor skills in lateralization uses

Correlational analysis showed that the locomotion categories involving the first step taken to perform an action were significantly correlated (Table 5), the stability categories involving turn direction were significantly correlated (Table 6), and the manipulation skills with upper and lower limbs were significantly correlated (Table 7).

**Table 5:** Significant correlations between specific motor skills involving locomotion skills.

		**Foot used to take the first step**	**First foot used to go up steps/stairs**	**Foot moved to regain balance**
Foot used to take the first step	Pearson correlation	1	0.469^**^	0.449^**^
	Significance (two-tailed)		0.000	0.000
	*N*	95	95	95
First foot used to go up steps/stairs	Pearson correlation	0.469^**^	1	0.368^**^
	Significance (two-tailed)	0.000		0.000
	*N*	95	95	95
Foot moved to regain balance	Pearson correlation	0.449^**^	0.368^**^	1
	Significance (two-tailed)	0.000	0.000	
	*N*	95	95	95

** *The correlation is significant at p < 0.01 (two-tailed)*.

**Table 6:** Significant correlations between specific motor skills involving stability skills.

		**Makes a full turn on both feet**	**Gets up from a chair and turns around to face the chair**	**Pivots (turns) on one foot from a standing position with feet parallel to each other**
Makes a full turn on both feet	Pearson correlation	1	0.370^**^	0.416^**^
	Significance (two-tailed)		0.000	0.000
	*N*	95	95	95
Gets up from a chair and turns around to face the chair	Pearson correlation	0.370^**^	1	0.411^**^
	Significance (two-tailed)	0.000		0.000
	*N*	95	95	95
Pivots (turns) on one foot from a standing position with feet parallel to each other	Pearson correlation	0.416^**^	0.411^**^	1
	Significance (two-tailed)	0.000	0.000	
	*N*	95	95	95

** *The correlation is significant at p < 0.01 (two-tailed)*.

**Table 7:** Significant correlations between specific motor skills involving manipulation skills.

		**Hand used to touch the object**	**Hand used to pick up the ball**	**Hand used to bounce the ball**	**Foot used to kick the ball**	**Foot that touches the ball first**
Hand used to touch the object	Pearson correlation	1	0.603^**^	0.390^**^	0.346^**^	0.239^*^
	Significance (two-tailed)		0.000	0.000	0.001	0.020
	*N*	95	95	95	95	95
Hand used to pick up the ball	Pearson correlation	0.603^**^	1	0.646^**^	0.235^*^	0.419^**^
	Significance (two-tailed)	0.000		0.000	0.022	0.000
	*N*	95	95	95	95	95
Hand used to bounce the ball	Pearson correlation	0.390^**^	0.646^**^	1	0.435^**^	0.715^**^
	Significance (two-tailed)	0.000	0.000		0.000	0.000
	*N*	95	95	95	95	95
Foot used to kick the ball	Pearson correlation	0.346^**^	0.235^*^	0.435^**^	1	0.748^**^
	Significance (two-tailed)	0.001	0.022	0.000		0.000
	*N*	95	95	95	95	95
Foot that touches the ball first	Pearson correlation	0.239^*^	0.419^**^	0.715^**^	0.748^**^	1
	Significance (two-tailed)	0.020	0.000	0.000	0.000	
	*N*	95	95	95	95	95

** *The correlation is significant at p < 0.01 (two-tailed)*.

* *The correlation is significant at p < 0.05 (two-tailed)*.

In relation to the fourth criterion—combined skills—Table 8 shows a significant correlation among the skills that required a change of spatial direction. In contrast, this correlation is not as evident for activities that required jumping. Additionally, the fourth MOTORLAT criterion differentiates athletes' uses of laterality by type of sport. The motor skills that best explain uses of laterality by sport are turn direction and jumps. The frequencies shown in Table 9 suggest that athletes in various sports—with the exception of gymnastics—tend to prefer performing turns to the left.

**Table 8:** Significant correlations between specific motor skills involving a change of direction.

		**Direction of rotation around head**	**Direction of rotation around waist**	**Direction of turn with feet parallel to each other**
Direction of rotation around head	Pearson correlation	1	0.525^**^	0.224^*^
	Significance (two-tailed)		0.000	0.029
	*N*	95	95	95
Direction of rotation around waist	Pearson correlation	0.525^**^	1	0.270^**^
	Significance (two-tailed)	0.000		0.008
	*N*	95	95	95
Direction of turn with feet parallel to each other	Pearson correlation	0.224^*^	0.270^**^	1
	Significance (two-tailed)	0.029	0.008	
	*N*	95	95	95

** *The correlation is significant at p < 0.01 (two-tailed)*.

* *The correlation is significant at p < 0.05 (two-tailed)*.

**Table 9:** Frequencies involving turn direction skills by sport.

		**Athletics**	**Gymnastics**	**Manipulation**	**Team sports**	**Total**
Makes a full turn on both feet	Right	6	9	1	15	31
	Left	11	8	9	36	64
Gets up from a chair and turns around to face the chair	Right	5	9	4	16	34
	Left	12	8	6	35	61
Pivots (turns) on one foot from a standing position with feet parallel to each other	Right	5	5	3	16	29
	Left	12	12	7	35	66
Total		17	17	10	51	95

### Spatial orientation and laterality profile

The spatial orientation strategies used by athletes after the first step to complete the PATHoops task were classified as follows:

1. Same way: The athlete goes to the same wing as the foot used in the first step (e.g., right-right).
2. Opposite way: The athlete goes to the opposite wing as the foot used in the first step (e.g., left-right).
3. Other: The athlete performs some other type of spatial orientation strategy, such as first walking forward across the hoops and omitting some of the lateral ones, or walking across the hoops anarchically, which results in mistakes such as failing to step in some hoops or stepping in some hoops more than once.

Table 10 shows that the most common spatial orientation strategy used by the athletes after the first step was “same way,” followed by “opposite way.”

**Table 10:** Contingency table of spatial orientation strategies used after the first step.

		**Same way**	**Opposite way**	**Other**	**Total**
First foot used to go up steps/stairs	Right	31	23	17	71
	Left	9	10	5	24
Makes a full turn on both feet	Right	13	11	7	31
	Left	27	22	15	64
Foot that touches the ball first	Right	35	28	20	83
	Left	5	4	2	11
	Ambidexterity	0	1	0	1
Hand used to touch the object	Right	36	29	21	86
	Left	4	4	1	9
Total		40	433	22	95

## Discussion

The aim of this study was to use the complementary tools of MOTORLAT and PATHoops to perform an objective analysis of young athletes' use of laterality in an increasingly complex range of motor skills and spatial orientation tasks involving a novel motor situation. To obtain a broad interpretation from the two instruments, we have conducted a contingency analysis to cross the limb dominance criteria from the MOTORLAT inventory and their relationships with the spatial orientation criteria from the PATHoops task. The discussion section is structured in the following sections: (a) Athletes' laterality profiles; (b) Laterality profiles and sport specialization; and (c) Spatial orientation and laterality profile (based on the findings from PATHoops crossed with the profiles obtained from MOTORLAT). Each section ends with clues about how coaches, educators and athletes can better understand how the laterality of the whole body and limbs underpins the diversity of motor skills used in sports and improve the performance of complex movements.

### Athletes' laterality profiles

Despite the anatomical symmetry of the body, humans exhibit a broad range of asymmetric usage of the limbs in the execution of motor actions (Palmer, 2004; for a review, see Brancucci et al., 2009). This evidence supports an integral perspective on the whole body that takes into account the contralateral synergy that combines postural-support and gestural-precision functions (Castañer et al., 2017a). We have therefore gone beyond the terms *handedness* and *footedness* as used in sports science (for a review, see Tran and Voracek, 2016), focusing instead on the concept of *motor laterality profile*. To move forward with this concept and offer MOTORLAT as a suitable inventory for assessing laterality, we based the tool on fundamental motor skills—locomotor, stability, and manipulation (Gallahue and Cleland-Donnelly, 2003; Castañer et al., 2009, 2015)—which have their roots in the phylogenetic contribution, display personalized ontogeny (Anderson et al., 2001; Salesse et al., 2005) and are geared toward adapting to multifaceted surroundings (Johnson, 2007) not only in sports but also in everyday life.

The MOTORLAT items used in this study allowed us to observe these contralateral uses, since the cluster analysis involving the three first criteria—locomotion, stability, and manipulation motor skills—reveal four clear motor laterality profiles: ambidexterity (1.1%), left laterality (2.6%), right laterality (3.72%), and mixed laterality (18%). Mixed laterality corresponds to athletes who perform locomotion and stability motor skills with the left lower limb and in a leftward direction but perform manipulation skills with the right upper and lower limbs.

As mentioned above, hand preference is a long-studied topic, including in the field of sports science. It has been suggested that left-handers may have an advantage over right-handers in various interactive sports, as demonstrated, for example, in our study of Lionel Messi's motor skill expertise in goal-scoring (Castañer et al., 2016a). However, as noted above, the left-footed Lionel Messi signs his contracts with his right hand. Even without knowing the full motor laterality profile of the best sportsman exhibiting mixed laterality, our results, obtained using the MOTORLAT combined motor skills criterion, differentiate athletes' uses of laterality by type of sport, showing a clear tendency toward mixed laterality profiles.

### Laterality profiles and sport specialization

The fourth MOTORLAT criterion—combined skills—is the best criterion for explaining the use of laterality in sports because the most complex movements tend to include the actions of turn direction and jumping. The results show that athletes in various sports perform turn direction mainly to the left. The results of a cluster analysis on jumping skills show that most athletes usually use their right hand to touch an elevated object, orienting their body to the left side. These results also fit with the cluster mentioned in the laterality profiles section, in which athletes with a mixed laterality profile (18%) perform locomotion and stability motor skills with the left lower limb and in a leftward direction but perform manipulation skills with the right upper and lower limbs.

These results clearly offer more evidence to support the argument that “postural support enables stasis and blocks movement, which allows the zone involved in gestural precision to execute the dynamics of the corresponding motor action” (Castañer et al., 2012a, p. 133). In another study by Castañer et al. (2017a), this quality was observed in Messi, who, with his back to the goal, would turn on his right leg, leaving his left leg to execute the goal-scoring action with greater precision. However, since laterality does not refer only to left-right preference (Velotta et al., 2011; McGrath et al., 2015) but also to how players orient their bodies spatially (Castañer et al., 2012a; Loffing et al., 2015), our study supports the notion that mixed laterality profiles enhance the performance of complex movements in athletes, adding value advantages in sport sciences (Tran and Voracek, 2016).

We therefore advise athletes, coaches and teachers that the successful use of certain patterns of mixed laterality promotes versatility of movement and could be used to enhance expertise in the performance of complex technical movements (Murgia et al., 2014; Schaefer, 2014). For example, stability skills are a versatile aspect because jumps, turns, balancing actions, and swinging actions serve to redistribute body weight, to play with gravity, or to prepare for or initiate the next move. Therefore, if an athlete uses his or her left leg for postural support to allow the right leg perform precise actions—such as those mentioned above—this contralateral use is the best option.

### Spatial orientation and laterality profile

The acquisition of spatial concepts (Pitchford et al., 2016) is a process directly related to uses of hemispheric dominance. The complex merging between hemispheric dominance and spatial orientation reinforces the framework integrating perception and action that was first addressed within the Theory of Event Coding (Hommel et al., 2001), which is related to assumptions such as spatial stimulus-response compatibility, sensorimotor synchronization, and ideomotor action. If hemispheric dominance is directly related to how one's body performs motor skills, having to manage the spatial orientation of the body is an allocentric point of view: “Spatial updating allows people to keep track of the self-to-object relations during movement” (Santoro et al., 2017). In PATHoops, participants are asked to perform a path by stepping in each of 14 hoops arranged on the floor, allowing researchers to observe their feet, their left-right preference and their spatial orientation. This task allowed us to achieve our objective of detecting spatial orientation from a novel motor situation that required participants to activate an ideomotor action as an empirical domain of the perception-action integration framework. It also fit with the assumption that the acquisition of locomotor skill is linked to developmental changes in an infant's ability to regulate posture on the basis of information available in patterns of optic flow (Anderson et al., 2001).

Our results show that the best strategies for the PATHoops task, after the first step, were “same way” (the athlete goes to the same wing as the foot used in the first step, e.g., right-right) followed by “opposite way” (the athlete goes to the opposite wing as the foot used in the first step, e.g., left-right). The most commonly used spatial orientation strategy was “same way,” followed by “opposite way,” in keeping with the assumption of Loffing et al. (2016) that actions unfolding in a horizontal direction in front of an observer's eyes are common in a variety of sports. These findings are consistent with the findings of previous research (Castañer et al., 2016a, 2017a).

We encourage athletes, coaches, and teachers to use tasks like PATHoops, which participants—not only athletes but also people of various ages and motor capabilities—must perform from both sides using locomotor skills. This guarantees a spontaneous stimulus-response, thereby avoiding previous automatic or rehearsed responses (Hommel et al., 2001; Castañer et al., 2010b, 2011, 2012b; Stöckel and Weigelt, 2012; Torrents et al., 2013). Despite involving the use of a fundamental and automatic locomotor skill—walking quickly—the novelty of the PATHoops task requires the use of multisensory information such as vestibular, visual, and kinesthetic information (Santoro et al., 2017). This could be suitable in decision-making, for example, when athlete must execute a feint or react to an object and decide which direction to take and, therefore, which foot to use to support his or her bodyweight and which foot to use to begin the movement.

## Conclusions and future lines of study

The objective of this study was to further our understanding of body laterality, taking into account the two main functions combined by the upper and lower limbs of the body—precision and support—as well as the spatial direction and orientation of the body. To achieve this objective, we used a combination of two instruments—the MOTORLAT inventory and the PATHoops task—to describe the “tapestry” of motor skills and contextual aspects that make up the singular style of each participant. In particular, spatial orientation and turning and jumping—which demand more complexity of movement—are described in this study. The 30 MOTORLAT items cover a range of movements from simple to complex motor skills, allowing experts to choose which ones might be of interest. The PATHoops task is a good complement for observing the spatial orientation strategies employed by participants. We consider that both instruments are a good fit for motor laterality studies because there is a need for deeper study of the motor skills underpinning the complex movements (Murgia et al., 2014; Schaefer, 2014) that athletes use in sports. As for the practical implications of this study, we would like to highlight the following:

- The capacity to detect laterality profiles defined by contralateral synergy (Castañer et al., 2012b) is an added value that makes it possible to optimize complex movements in sports that are traditionally more focused on tactical and technical analysis.
- Athletes, coaches, and teachers in the field of physical activity and sports can use these findings to implement tasks related to acquiring skills and improving the efficacy of complex movements.
- Young athletes have a broad workout background in terms of bilateral practice schedules, whose effects in relation to brain lateralization (Stöckel and Weigelt, 2012) could be of interest with regard to neurocognition approaches.
- In this study, we focused mainly on motor laterality. This research can be extended through deeper studies of perceptive laterality in order to strengthen the evidence on non-integral lateralization and the implications of mixed laterality in body movements, with the aim of better discriminating between the successful and unsuccessful performance of complex movements.

With this study, we hope to have contributed to extending the research on motor laterality and spatial orientation. We agree with Loffing et al. (2016) that mixed laterality in sports has become a recent focus of research and requires more extensive study in order to explore contralateral functions in motor skill acquisition, technique learning, and dealing with complex movements in order to optimize performance in sports (Murgia et al., 2014; Schaefer, 2014).

From a methodological point of view, the use of two instruments that combine bilateral limb usages and spatial orientation for a broader assessment of laterality is in keeping with the mixed methods approach, which combines techniques to offer a better way of achieving objectives (e.g., Creswell, 2015). Given that we generally conduct our research using mixed method designs that combine qualitative and quantitative data and analytical techniques—such as triangulation, embedded, or explanatory designs—in a parallel or sequential way (Anguera et al., 2014, 2017), we encourage researchers to move beyond the use of a single instrument and embrace the combination of multiple instruments to find a better way of achieving research objectives.

## Author contributions

MC developed the project, supervised the design of the study, the method section, and the drafting of the manuscript. SP was responsible for the review of the literature. OC was responsible for the critical revision of the content. RH performed the validation of the laterality inventory and the drafting of the manuscript. JA was responsible for the drafting of the manuscript and collected and codified the data. QP supervised the drafting of the manuscript. All authors approved the final, submitted version of the manuscript.

### Conflict of interest statement

The authors declare that the research was conducted in the absence of any commercial or financial relationships that could be construed as a potential conflict of interest.
